# Association between COVID-19 diagnosis and severe mental health symptoms in adolescents in southern Brazil

**DOI:** 10.1590/1984-0462/2026/44/2025016

**Published:** 2026-01-19

**Authors:** Yohana Pereira Vieira, Eduardo Gauze Alexandrino, Rinelly Pazinato Dutra, Yasmin Marques Castro, Vicente Gabriel Winck Mattos, Andressa Munhoz Sá, Samuel de Carvalho Dumith

**Affiliations:** a Universidade Federal do Rio Grande, Rio Grande, RS, Brazil. Universidade Federal do Rio Grande Rio Grande RS Brazil

**Keywords:** Depression, Anxiety, Adolescent, Mental health, Post-COVID-19 syndrome, Depressão;, Ansiedade, Adolescentes, Saúde mental, Síndrome pós-COVID-19

## Abstract

**Objective:**

To evaluate the association between self-reported COVID-19 and the occurrence of severe mental health symptoms such as anxiety, stress, and depression among high school students in southern Brazil.

**Methods:**

Cross-sectional study with high school adolescents from the Instituto Federal do Rio Grande do Sul, Brazil. The outcomes were anxiety, depression, and severe stress assessed through the “Adolescent Anxiety, Depression, and Stress Scale” (DASS-21), and the diagnosis of COVID-19 was obtained through self-report. Descriptive and bivariate analyses were performed. Poisson regression with robust variance adjustment and multinomial logistic regression were used to verify associations.

**Results:**

Of the 462 participants, 16.6, 15.6, and 9.9% reported severe symptoms of depression, anxiety, and stress, respectively, and 41% reported a diagnosis of COVID-19. In the adjusted analysis, adolescents diagnosed with COVID-19 were more likely to have severe anxiety symptoms (prevalence ratio — PR 2.12; 95% confidence interval — 95%CI 1.35–3.33) and three times more likely (OR 2.98; 95%CI 1.35–6.56) to develop severe symptoms of two of the three disorders investigated.

**Conclusions:**

Adolescents who reported a COVID-19 diagnosis experienced worse mental health outcomes, with associations observed for severe anxiety and for the presence of multiple severe symptoms of depression, anxiety, or stress. These findings highlight the need for further clinical and epidemiological research to better understand and address the impact of COVID-19 on adolescent mental health.

## RESUMO

**Objetivo:**

Verificar a associação entre o autorrelato de COVID-19 com sintomatologias graves de ansiedade, estresse e depressão em escolares do sul do Brasil.

**Métodos:**

Estudo transversal com adolescentes do ensino médio do Instituto Federal do Rio Grande do Sul em Rio Grande, Brasil. Os desfechos foram ansiedade, depressão e estresse grave avaliados por meio da “Escala de Ansiedade, Depressão e Estresse para Adolescentes” (DASS-21), e o diagnóstico de COVID-19 foi obtido por meio do autorrelato. Foram realizadas análises descritivas e bivariadas e, para verificar as associações, foi utilizada a regressão de Poisson, com ajuste robusto da variância e regressão logística multinomial.

**Resultados:**

Dos 462 participantes 16.6, 15.6 e 9.9% relataram sintomas graves de depressão, ansiedade e estresse, respectivamente, e 41% relataram diagnóstico de COVID-19. Na análise ajustada, os adolescentes com diagnóstico de COVID-19 tiveram maior probabilidade de apresentar sintomas graves de ansiedade (razão de prevalência — RP 2.12; intervalo de confiança de 95% — IC95% 1.35–3.33) e três vezes mais chances (OR 2.98; IC95% 1.35–6.56) de desenvolver sintomas graves de dois dos três transtornos investigados.

**Conclusões:**

Adolescentes que relataram diagnóstico de COVID-19 apresentaram piores desfechos de saúde mental, com associações observadas para ansiedade grave e para a presença de múltiplos sintomas graves de depressão, ansiedade ou estresse. Esses achados destacam a necessidade de mais pesquisas clínicas e epidemiológicas para melhor compreender e abordar o impacto da COVID-19 na saúde mental dos adolescentes.

## INTRODUCTION

Coronavirus disease 19 (COVID-19) is an infectious disease caused by the Severe Acute Respiratory Syndrome Coronavirus 2 (SARS-CoV-2) virus. The number of reported cases in Brazil is 37,579,028, with 702,654 deaths.^1^ After the acute infection, a new condition arises where individuals report long-term symptoms, between four to 12 weeks post-infection, known as long COVID.^2,3^ A study in England indicated a prevalence of long COVID in adolescents aged 12–16 years at 0.51% and 17–24 years at 1.21%.^2^ Other meta-analyses have indicated that, in people under 18 years old, the most prevalent symptoms are headache, muscle pain, and neuropsychiatric symptoms such as sleep disturbances, fatigue, depression, anxiety, and post-traumatic stress disorder,^4,5^ with neurological and psychiatric symptoms being more prevalent in severe cases.^5,6^

Adolescents are considered a vulnerable population regarding mental health outcomes and require both comprehensive and continuous care, as well as the establishment of a consistent support network, particularly in this pandemic context.^7^ This is because, in general, the COVID-19 pandemic has brought numerous implications to adolescents’ lives. Notable among these are the sudden suspension of classes and home confinement due to social distancing guidelines, as well as the occurrence of acute and chronic stress, high levels of concern for their health and that of their families, and experiences of grief, sadness, and fear.^8,9^ All these aspects have led to a significant increase in the prevalence of mental health symptoms such as anxiety and depression^9,10,11^ and even to the worsening of more severe disorders across various age groups.^7,12^

Additionally, those experiencing long-term COVID-19 have reported clinical symptoms that resemble general mental health somatic symptoms.^5,13^ Although self-reported symptoms cannot be equated with the diagnosis of psychopathologies, the increase in these symptoms in this age group is concerning.^9,14^

While stress, anxiety, and depression are symptoms present in the spectrum of long-term COVID-19 in adolescentes,^15,16^ a recent study found no significant differences between SARS-CoV-2 infected and non-infected adolescents regarding the occurrence of mental health symptoms.^14^It is important to note, however, that most existing studies address the effect of the pandemic context without explicitly examining the relationship between SARS-CoV-2 infection and adolescent mental health^7,8,11,12^ or they do not distinguish analyses by age group for children or adolescents.^5,17^ These aspects highlight an essential gap in the scientific literature regarding specific investigations on the effect of SARS-CoV-2 infection and the occurrence of long-term COVID-19 on adolescent mental health.^14^

Given the aspects presented and the importance of investigating the presence of psychological symptoms and the occurrence of psychiatric disorders among students who have had long COVID,^7,11,13,18^ the objective of the present study was to evaluate the association between self-reported COVID-19 and the occurrence of severe mental health symptoms such as anxiety, stress, and depression among adolescents high school students in southern Brazil.

## METHOD

This is a cross-sectional, census-type study of high school students at the Instituto Federal do Rio Grande do Sul (IFRS), Rio Grande campus, state of Rio Grande do Sul, Brazil. The study participants were all students enrolled in high school at IFRS, Rio Grande campus, during the second semester of 2022, totaling 510 enrollments from the first to the third year. Students with physical or cognitive conditions that prevented them from completing the questionnaire were excluded from the study.

The study was conducted at IFRS due to its accessibility and institutional support, which facilitated the data collection process in an ethical and organized manner. The IFRS is a public federal education institution that offers technical and higher education programs in multiple campuses across the state. The Rio Grande campus has a significant number of adolescents enrolled in integrated and regular high school programs, making it a suitable setting for reaching the study’s target population.

For data collection, initial contact was made with the institution’s administration and subsequently with the classes to present the research and read the informed consent form (ICF) for minors under 18 years old and the assent form (AF). Data collection took place in October 2022 through self-administered questionnaires on individual tablets. The data collection and questionnaire administration was conducted by interviewers (eight postgraduate students and two undergraduate students in scientific initiation) who had been previously trained and were responsible for providing instructions, identifying students, and distributing the tablets.

Students who chose not to participate in the research were considered refusals, and those who could not be found after four visits for data collection were considered losses. The time to complete the questionnaire ranged from 20 to 30 minutes. The data were stored on the Research Electronic Data Capture — REDCap^®^ platform.

In this study, the outcomes investigated were severe anxiety, depression, and stress, assessed through the “Depression, Anxiety, and Stress Scale for Adolescents” (DASS-21).^19^ The DASS-21 is a self-administered Likert-type scale, where the three domains are determined by summing the scores of the 21 items. Each question can range from 0 to 3 points, and, for interpretation, the total score corresponding to the responses was used: 0 (did not happen to me this week), 1 (happened to me a few times this week), 2 (happened to me most of the week), 3 (happened to me most of the time this week).

For each mental health domain assessed by the DASS-21 (depression, anxiety, and stress), a dichotomous variable was created indicating the presence (“yes”) or absence (“no”) of at least one symptom classified as severe or extremely severe, according to the scoring criteria of the instrument. Thus, in this study, severe symptoms refer specifically to those that met these cutoff points. In addition, a composite variable named “severe symptoms of depression/anxiety/stress” was created to indicate whether the adolescent presented at least one severe or extremely severe symptom in any of the three domains.

A mental health score (mental score) was also calculated to represent the number of domains in which each adolescent exhibited severe or extremely severe symptoms. This score ranged from 0 to 3, where 0 indicated no severe symptoms in any domain, 1 indicated severe symptoms in one domain, 2 in two domains, and 3 in all three domains. This variable was used as an ordinal indicator of overall mental health symptom severity.

COVID -19 exposure was assessed through the question “Have you ever had COVID-9?” with response options “no” and “yes.” The adjustment variables were sex (male/female), age (15/16/17/18 years), skin color or race (white/black, yellow, or indigenous), and physical activity (<150 min/week = no/ ≥150 min/week = yes). The REDCap^®^ database, containing information from the self-administered questionnaires, was exported to the STATA 15.1 statistical package, where the study analyses were conducted. First, the prevalence of outcomes was described according to the absence and presence of COVID-19. Then, crude and adjusted analyses were performed to identify the association between dichotomous mental health outcomes (severe anxiety, depression, and stress) and COVID-19 using Poisson regression with robust variance adjustment, presenting the prevalence ratio (PR) and 95% confidence intervals (95% CI), for the association between the polytomous mental health outcome (mental health score) and COVID-19, crude and adjusted analyses were performed using multinomial logistic regression. p<0.05 were considered statistically significant for two-tailed tests.

This research project was approved by the Research Ethics Committee of the Federal University of Rio Grande under opinion No. 3,824,558, Certificate of Presentation for Ethical Consideration (CAAE): 26359019.00000.5324. All individuals over 18 years old or their guardians (if under 18) who agreed to participate in the research must sign the ICF. Additionally, before completing the questionnaire, all participants had to sign the AF to participate in the research. The reporting of this study followed the Strengthening the Reporting of Observational Studies in Epidemiology (STROBE) Statement guidelines.

## RESULTS

**Figure 1.**
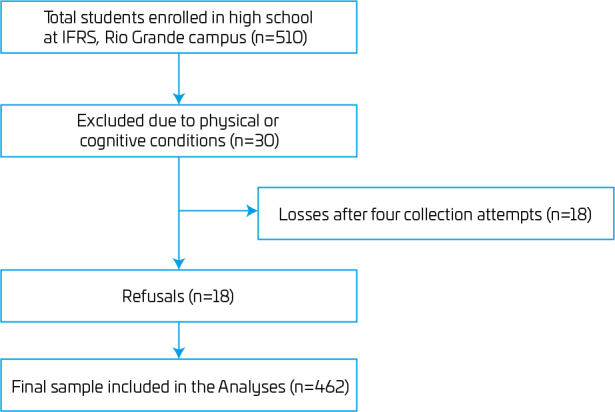
Flowchart of recruitment of students of the Instituto Federal do Rio Grande do Sul, Rio Grande campus, Rio Grande do Sul, Brazil, 2022.

Among the 510 enrolled students, 30 were not regularly attending classes, making 480 students eligible for the study. Of these, 18 students were absent during the month of data collection, yielding a response rate of 91% (Figure 1). Of the 462 study participants, 41% (95%CI 36.5–45.7) reported COVID-19 diagnosis. Regarding the sample characteristics, more than half were male (54.3%), 52.6% were 18 years old at the time of data collection, and the majority reported white skin color (76%) (Table 1).

IFRS: Instituto Federal do Rio Grande do Sul.

**Table 1.** Characteristics of the sample of high school students at the Instituto Federal do Rio Grande do Sul, Rio Grande campus, 2022 (n=462).

Variables	n	% 1
Sex		
Masculine	251	54.3
Feminine	211	45.7
Age (years)		
15	81	15.6
16	67	15.4
17	102	23.4
18	101	23.2
19	85	19.5
Skin color		
White	333	76.0
Black/Brown/Indigenous	101	24.0
Physically active		
No	335	77.5
Yes	97	22.5

Notes: Physically active: ≥150 min/week.

As for the mental health outcomes assessed, 16.6% (95%CI 13.4–20.4), 15.5% (95%CI 12.4–19.3), and 9.9% (95%CI 7.4–13.1) reported severe symptoms of depression, anxiety, and stress, respectively. Table 2 shows that the prevalence of worse mental health outcomes was higher among students who reported a COVID-19 diagnosis. Severe anxiety symptoms were more than double among students who had COVID-19 (10.2% versus 23.4%). Students who reported a COVID-19 diagnosis had more severe depression symptoms (20.3%) and severe stress (12%) compared to those who did not report a positive diagnosis. When severe symptoms were grouped, the presence of at least one of the three studied health conditions was almost double among students who had COVID-19 (17.7% versus 30.5%).

**Table 2.** Prevalence of outcomes according to absence and presence of COVID-19 among high school students at the Instituto Federal do Rio Grande do Sul, Rio Grande campus, 2022 (n=462).

Variables	Total	Without COVID-19	With COVID-19
	% (95%CI)	% (95%CI)	% (95%CI)
Severe anxiety			
No	84.5 (80.7–87.6)	89.8 (85.4–92.9)	76.6 (69.8–82.4)
Yes	15.5 (12.4–19.3)	10.2 (7.1–14.6)	23.4 (17.6–30.2)
Severe depression			
No	83.4 (79.6–86.6)	85.9 (81.1–89.7)	79.7 (73.0–85.0)
Yes	16.6 (13.4–20.4)	14.1 (10.3–18.9)	20.3 (15.0–27.0)
Severe stress			
No	90.1 (86.9–92.6)	91.8 (87.7–94.6)	87.5 (81.7–91.7)
Yes	9.9 (7.4–13.1)	8.2 (5.4–12.3)	12.5 (8.3–18.3)
Severe symptoms of depression/anxiety/stress			
No	77.2 (72.9–80.9)	82.3 (77.1–86.5)	69.5 (62.3–76.0)
Yes	22.8 (19.1–27.0)	17.7 (13.5–22.9)	30.5 (24.0–37.7)
Mental score			
0	77.2 (72.9–80.9)	82.2 (77.1–86.5)	69.5 (62.3–76.0)
1	10.2 (7.7–13.5)	8.7 (5.8–12.8)	12.7 (8.4–18.5)
2	7.0 (4.9–9.8)	4.4 (2.4–7.7)	10.9 (7.1–16.5)
3	5.6 (3.8–8.2)	4.7 (2.7–8.2)	6.9 (3.9–11.8)

Note: Mental score ranges from 0 to 3 and indicates the number of domains (depression, anxiety, stress) in which the adolescent exhibited severe or extremely severe symptoms.

In the adjusted analysis, students who reported COVID-19 were 2.12 times more likely to have severe anxiety symptoms compared to those who did not have this condition (PR 2.12; 95%CI 1.35–3.33) and 1.60 times more likely to have at least one of the three severe symptoms of anxiety, depression, or stress (PR 1.60; 95%CI 1.13–2.27). Regarding the mental score of symptoms, students who had COVID-19 were three times more likely to manifest two severe symptoms of anxiety, depression, or stress (OR 2.98; 95%CI 1.35–6.56) (Table 3).

**Table 3.** Analysis of the association between selfreported COVID-19 and mental health outcomes among high school students at the Instituto Federal do Rio Grande do Sul, Rio Grande campus, 2022 (n= 462).

Outcomes	Crude analysis	Adjusted analysis
	PR (95%CI)	PR (95%CI)
Severe anxiety		
No	1	1
Yes	**2.28 (1.45–3.58)**	**2.12 (1.35–3.33)**
Severe depression		
No	1	1
Yes	1.44 (0.95–2.20)	1.38 (0.89–2.12)
Severe stress		
No	1	1
Yes	1.52 (0.86–2.69)	1.41 (0.78–2.54)
Severe symptoms of depression/anxiety/stress		
No	1	
Yes	**1.72 (1.21–2.43)**	**1.60 (1.13–2.27)**
Mental score*	**OR (95%CI)**	
0	1	1
1	1.72 (0.92–3.25)	1.62 (0;85–3.10)
2	**2.98 (1.37–6.48)**	**2.98 (1.35–6.56)**
3	1.72 (0.75–3.96)	1.46 (0.59–3.62)

*Multinomial regression.

PR: prevalence ratio; OR: odds ratio. 95%CI: 95% confidence interval. Note: Adjusted for sex, age, skin color or race, and physical activity. Poisson regression with robust adjustment for variance. Mental score ranges from 0 to 3 and indicates the number of domains (depression, anxiety, stress) in which the adolescent exhibited severe or extremely severe symptoms. Bold indicates statistically significant p-values.

## DISCUSSION

This study analyzed the association between severe symptoms of anxiety, depression, and stress with self-reported COVID-19 among 462 high school students in southern Brazil. The results indicated that 41% of the students reported a COVID-19 diagnosis, and there was a significant association between having had the disease and the manifestation of severe anxiety symptoms, as well as the presence of at least one severe symptom of anxiety, depression, or stress. Adolescents who reported being diagnosed with COVID-19 had higher prevalences of severe stress and depression and were more likely to develop severe anxiety symptoms compared to those who were not infected. Additionally, adolescents who had COVID-19 exhibited a more significant burden of associated mental health symptoms, manifesting severe symptoms in two of the three mental health conditions assessed.

Systematic reviews conducted during the pandemic period showed concerning mental health trends across various populations. Among the school population in the first year of the pandemic, a meta-analysis of 29 studies collected data from 80,879 young people worldwide. The authors found a prevalence of 25.2% for depression and 20.5% for anxiety and highlighted that the prevalence of depression and anxiety symptoms during COVID-19 doubled compared to pre-pandemic estimates.^11^ Later, in 2022, a large meta-analysis that evaluated 1,389,447 children and adolescents in 191 studies found a prevalence of 31% for depressive symptoms and 31% for anxiety symptoms among the under-18 population during the pandemic.^6^

Even after three years of the pandemic, children and adolescents represent a minority of COVID-19 cases, and the long-term effects on these age groups remain conflicting and far from conclusive.^16,20^ Adults who had psychiatric disorders in childhood or adolescence are at increased risk of developing substance dependence or having difficulty forming lasting social bonds.^21^

The prevalence of worse mental health outcomes among Brazilian schoolchildren who had COVID-19 observed in this study serves as a warning of the need for early detection and timely intervention in the school-age population. This data, combined with the natural history of psychiatric disorders and high prevalences observed in meta-analyses, indicates a possible increase in mental disorders among young people in Brazil after the pandemic period, making it essential for educational and health managers to maintain vigilant oversight. Other studies highlight the negative mental health impacts on infected children and adolescents. Lopez-Leon et al.^17^indicated a prevalence of 16.5% for mood change symptoms among adolescents with COVID-19 after 12 weeks of infection. Similarly, the review by Zawilska and Kuczynska^16^ observed the possibility of neuropsychiatric impairment in children and adolescents after 12 weeks of recovery from infection. Önder et al.^22^ also pointed out that having a more severe infection (being hospitalized for COVID-19) could increase the risk of children developing mental disorders, including anxiety and depression. Another study suggests that the impact on neuropsychiatric and somatic functions of long COVID can also be observed in pediatric patients.^20^

It is noteworthy that no peer-reviewed studies have been found so far that evaluate the impact of SARS-CoV-2 infection on mental health outcomes in the Brazilian school environment, as research has primarily focused on university students, teachers, and healthcare professionals. In addition to the increased risk of developing psychopathological syndromes, the infection is associated with the syndromic worsening of anxiety in adolescents who already had these conditions before the infection.^23^ The COVID-19 pandemic resulted in an abrupt change in people’s lifestyles, with isolation used both as a preventive measure and to prevent the spread of the virus and the fear of an unknown disease, which can be potent triggers for negative mental symptoms.^24^

In this study, 12.6% of the students had severe symptoms of at least two disorders. However, psychiatric comorbidities seem to be not uncommon, with estimates suggesting that around 40% of adolescents diagnosed with one mental disorder also suffer from another disorder.^25,26^ Depressive and anxious symptoms, in particular, coexist from adolescence to adulthood. The co-occurrence of these psychopathologies can amplify the negative impact on adolescents’ health, resulting in more significant deterioration of mental health, lower quality of life, and higher risk of long-term health problems.^27,28^

The mechanisms of SARS-CoV-2 remain poorly understood in terms of its neuropsychiatric involvement. Some possibilities being investigated are that the virus may directly affect nervous tissue, as its neuroinvasive solid potential can provoke a systemic inflammatory response, vascular effects, and pro-thrombotic effects in the central nervous system or vasculature of the peripheral nervous system.^18^

Regarding COVID-19, there are still gaps to be filled about the association between the occurrence of the disease and the aggregation of severe psychiatric symptoms, indicating the need for studies that delve deeper into these aspects. However, existing evidence points out that neuropsychiatric manifestations such as depression, anxiety, and post-traumatic stress are present in patients with long COVID. Factors such as disease severity, the existence of comorbidities, a history of mental disorders, and elevated levels of inflammatory markers are associated with a higher risk. However, more research is needed to determine cause-and-effect relationships.^29^ Furthermore, considering the pandemic context in general, it is observed that older age among children and adolescents, as well as being female, seem to be positively associated with the overall burden of mental health problems in this population.^30^

As for the strengths of this study, first, it should be noted that it presents COVID-19 data and mental health symptoms from a population of adolescents in Brazil, an age group for which few published studies exist. Second, the mental health variables were assessed through a questionnaire tested and validated in the literature, specifically for adolescents (DASS-21). However, the results of this study should be interpreted considering its limitations. First, the COVID-19 diagnosis was obtained through self-report, which may have caused recall bias. Furthermore, as this is a cross-sectional study, it is not possible to establish causality in the associations found, as it is not possible to know if the adolescents already had mental health problems before SARS-CoV-2 infection. Moreover, they were not asked about the presence of mental health issues prior to the pandemic. It is suggested that longitudinal studies be conducted in the future to monitor mental health symptoms of long COVID in the adolescent population.

In addition, caution is needed when generalising these findings. The study was conducted in a single educational institution in southern Brazil, which may not fully represent the diversity of adolescents in other regions or educational contexts. Therefore, the external validity of the results is limited, and replication in broader and more heterogeneous adolescent populations is necessary to strengthen the generalisability of the conclusions.

Adolescents who reported a COVID-19 diagnosis experienced worse mental health outcomes. There was an association between adolescents who reported COVID-19 and severe anxiety, as well as at least one or two severe symptoms of depression, anxiety, or stress. However, there are still significant gaps in the literature regarding the multidisciplinary therapeutic management of long COVID, particularly in the pediatric population. In this regard, more in-depth clinical and epidemiological investigations are needed to understand better the complex relationship between mental disorder symptoms and a COVID-19 diagnosis.

Psychiatric symptoms appear to be a highly prevalent feature of long COVID. The data from this study may contribute to future research on the epidemiology, treatment, and underlying mechanisms of psychiatric symptoms in long COVID, aiding in the improved clinical and preventive multidisciplinary management of this condition in the adolescent population.

## Data Availability

The database that originated the article is available with the corresponding author.
